# De novo lipogenesis in the liver in health and disease: more than just a shunting yard for glucose

**DOI:** 10.1111/brv.12178

**Published:** 2015-03-04

**Authors:** Francis W. B. Sanders, Julian L. Griffin

**Affiliations:** ^1^ MRC Human Nutrition Research, Elsie Widdowson Laboratory120 Fulbourn RoadCambridge CB1 9NLU.K.; ^2^ The Department of BiochemistryUniversity of CambridgeTennis Court RoadCambridge CB2 1GAU.K.

**Keywords:** de novo lipogenesis (DNL), non‐alcoholic fatty liver disease (NAFLD), fructose, liver, selective insulin resistance

## Abstract

Hepatic de novo lipogenesis (DNL) is the biochemical process of synthesising fatty acids from acetyl‐CoA subunits that are produced from a number of different pathways within the cell, most commonly carbohydrate catabolism. In addition to glucose which most commonly supplies carbon units for DNL, fructose is also a profoundly lipogenic substrate that can drive DNL, important when considering the increasing use of fructose in corn syrup as a sweetener. In the context of disease, DNL is thought to contribute to the pathogenesis of non‐alcoholic fatty liver disease, a common condition often associated with the metabolic syndrome and consequent insulin resistance. Whether DNL plays a significant role in the pathogenesis of insulin resistance is yet to be fully elucidated, but it may be that the prevalent products of this synthetic process induce some aspect of hepatic insulin resistance.

## I. INTRODUCTION

Hepatic *de novo* lipogenesis (DNL) is a fundamental biosynthetic pathway within the liver, contributing to the lipids that are stored and secreted by hepatocytes (Jensen‐Urstad & Semenkovich, 2012). This process is an extension of the complex metabolic networks at play within the liver, and is provided with substrate primarily through glycolysis and the metabolism of carbohydrates. Therefore, a high‐carbohydrate diet can prime the DNL pathway with a large substrate load and increase rates of DNL (Schwarz *et al*., 2003). Importantly this leads to an accumulation of DNL products, fatty acyl chains linked to coenzyme A, which can be incorporated into a plethora of lipid species. These lipids may then have further metabolic functions, which in turn may be deleterious in cases of elevated DNL.

DNL has been suggested to be abnormally increased in and contribute to the pathogenesis of non‐alcoholic fatty liver disease (NAFLD) (Donnelly *et al*., 2005), a highly prevalent metabolic disease that is linked to the development of type 2 diabetes mellitus (T2DM). DNL is also increased under conditions of insulin resistance (Ameer *et al*., 2014), and thus knowing the rate of DNL in an individual may benefit the clinician as it would provide early warning of the possible development of T2DM. This is especially important as recent evidence suggests that ‘prediabetes’, a state of mildly elevated blood glucose, has increased significantly from 11.6% in 2003 to 35.3% in 2011 amongst adults in England (Mainous *et al*., 2014). Therefore it may be crucial to understand and map the full spectrum of metabolic disturbances associated with insulin resistance, including rates of DNL.

## II. THE BIOCHEMICAL PROCESS OF DNL

**Figure 1:**
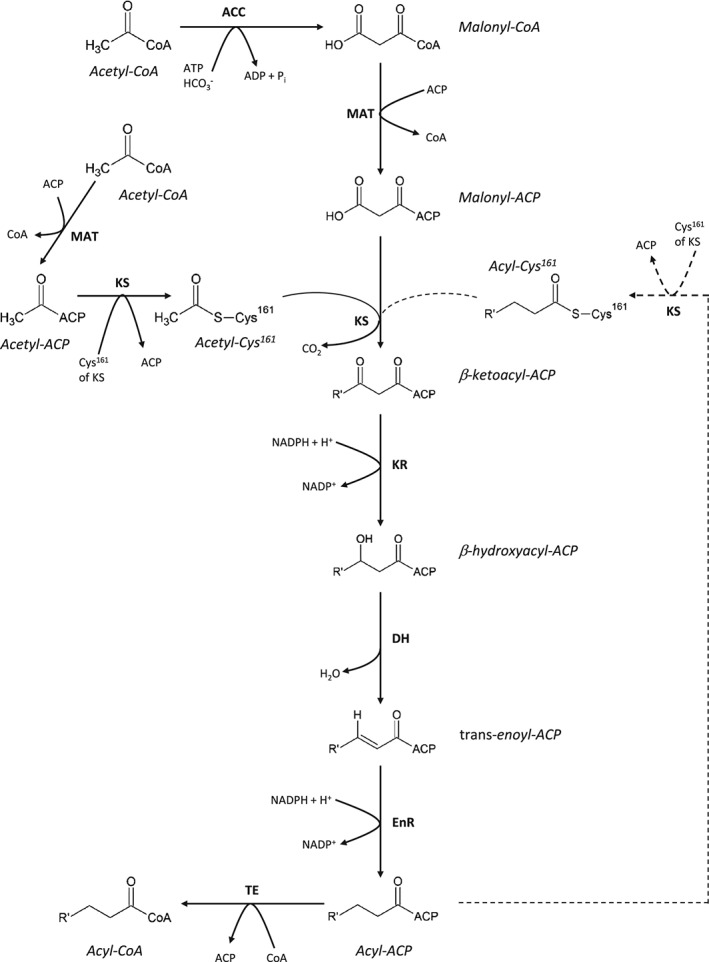
De novo lipogenesis. Malonyl‐CoA, as the additive monomer for DNL is generated from acetyl‐CoA under the catalytic activity of ACC. The acetyl and malonyl substrates for DNL are transferred to ACP. The acetyl‐CoA is then bound to the Cys^161^ of KS, followed by the decarboxylative condensation of acetyl‐CoA and malonyl‐CoA, forming β‐ketoacyl‐ACP catalysed by KS. The β‐ketone group of β‐ketoacyl‐ACP is reduced using the cosubstrate NADPH under the catalysis of KR, generating a β‐hydroxyacyl‐ACP intermediate. This reaction is followed by dehydration of the β‐carbon, producing a trans‐enoyl‐ACP intermediate. The trans double bond between the α‐ and β‐carbons is reduced under the catalytic action of EnR, using NAPDH as a substrate, forming acyl‐ACP. This can re‐enter the cycle to act as a substrate for KS, or when the R′ carbon chain is of sufficient length, the ACP is replaced with CoA and the acyl‐CoA released from FAS as TE is used to enzymatically cleave the acyl product from ACP. ACP, DH, EnR, KS, MAT and TE are all part of the multifunctional FAS. ACC, acetyl‐CoA carboxylase; ACP, acyl carrier protein; CoA, coenzyme A; DH, dehydratase; DNL, de novo lipogenesis; EnR, enoyl‐reductase; FAS, fatty acid synthase; KR, β‐ketoreductase; KS, β‐ketoacyl synthase; NADP^+^, nicotine adenine dinucleotide phosphate; NADPH, reduced form of NADP^+^; MAT, malonyl/acetyl transaferase; TE, thioesterase.

DNL is the synthesis of fatty acid (FA) chains from acetyl‐CoA subunits produced during glycolysis (Smith & Tsai, 2007) and these can undergo subsequent condensation with a glycerol backbone (Coleman & Lee, 2004) (Fig. 1).

The reaction mechanism commences with the production of malonyl‐CoA from an acetyl‐CoA precursor, under the regulated catalytic activity of acetyl‐CoA carboxylase (ACC) (Bianchi et al., 1990). The malonyl‐CoA is transferred to the prosthetic phosphopantetheine group of acyl carrier protein (ACP) (Majerus, Alberts & Vagelos, 1964), a domain of the type I fatty acid synthase complex (FAS) (Brindley, Matsumura & Bloch, 1969; Smith, 1994), with subsequent release of the coenzyme A carrier, catalysed by the activity of the malonyl/acetyl transferase (MAT) site of mammalian FAS (Mikkelsen et al., 1985). The prosthetic phosphopantetheine arm of ACP thus shuttles the elongating FA chain to the various catalytic centres in the active site cleft of FAS, aided by the rotation of FAS (Wakil, 1989; Smith & Tsai, 2007; Maier, Leibundgut & Ban, 2008).

The ACP‐bound malonyl moiety acts as the additive monomer for the elongation of the substrate acyl chain. Initially this is an acetyl unit bound to the thiol group of cysteine (Cys^161^) at the β‐ketoacyl synthase (KS) active site (Witkowski, Joshi & Smith, 1997). The malonyl moiety undergoes decarboxylative condensation with an acetyl moiety, as elucidated in Fig. 1 (von Wettstein‐Knowles et al., 2006). ACP is then bound to a β‐ketoacyl intermediate.

ACP shuttles the β‐ketoacyl intermediate to the NADPH‐dependent β‐ketoreductase (KR) active site (Chang & Hammes, 1990). The ketone of the β‐carbon is reduced, generating a hydroxyl group. This is followed by sequential dehydration, by the dehydratase (DH) active site, and further reduction by NADPH‐dependent enoyl‐reductase (EnR) (Wakil, 1989; Chang & Hammes, 1990; Smith & Tsai, 2007). This generates a saturated acyl chain elongated by two carbon groups, which can act as the substrate for the next round of elongation as it binds the thiol‐group of the cysteine at the catalytic site of KS.

The elongation ceases at the 16‐ or 18‐carbon stage (Foster & Bloom, 1963; Carey, Dils & Hansen, 1970) with release of palmitic acid or stearic acid from ACP via activity of the thioesterase (TE) domain of FAS (Chakravarty et al., 2004; Smith & Tsai, 2007). The specificity of FAS TE for 16‐carbon acyl dictates the length of the FAs released in vitro as there is a rapid decline of TE activity for chain lengths less than 14‐carbons (Lin & Smith, 1978; Chakravarty et al., 2004), as it will not access the catalytic core of the domain, and greater than 18‐carbons as it may not be accommodated by the binding groove of TE (Chakravarty et al., 2004). The termination of chain elongation at this 16‐ carbon stage is further promoted due to acyl chains of this length or longer not readily transferring to the thiol‐group of the active‐site cysteine of KS (Witkowski et al., 1997). The incorporation of stable‐isotope‐labelled precursors into palmitate in humans, and tritium from ^3^H_2_O in rat liver also supports palmitate as the major product of DNL (Foster & Bloom, 1963; Hellerstein et al., 1991; Murphy, 2006).

**Figure 2:**
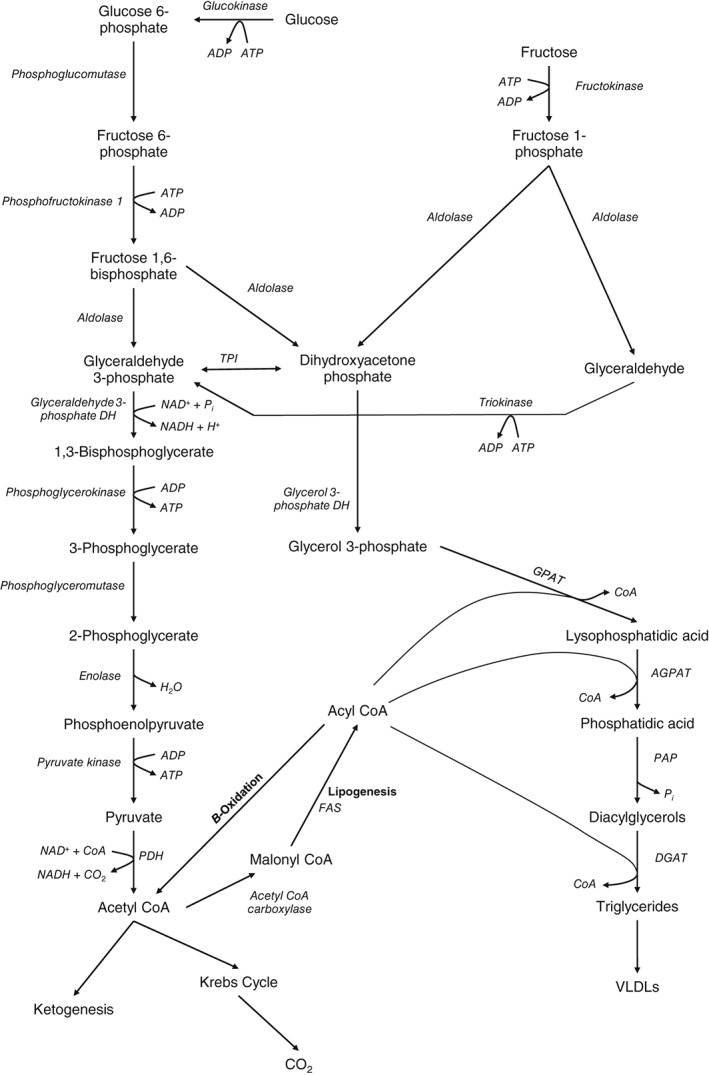
Hepatic glycolysis, fructolysis and triglyceride synthesis. Glucose undergoes the multistep process of glycolysis to generate pyruvate which is subsequently utilised for a range of cellular metabolic processes. This includes complete oxidative catabolism to carbon dioxide, lipogenesis under the catalysis of fatty acid synthase (FAS), and ketogenesis to generate ketone bodies for oxidative metabolic fuel. The fructolytic pathway feeds into the glycolytic pathway at the level of the trioses: dihydroxyacetone phosphate and glyceraldehyde 3‐phosphate. This thus bypasses the crucial regulatory steps in liver glycolytic metabolism, whereby glucokinase and phosphofructokinase‐1 are controlled by the metabolic milieu in coordination with hormonal effectors. This allows for the production of acetyl‐CoA from fructose to continue without regulation by insulin, and hence promote lipogenesis, and subsequent triglyceride (TG) synthesis from the acyl‐coenzyme A (CoA) product. This involves catalysis of progressive acylation of a glycerol‐phosphate backbone by glycerol‐phosphate acyl transferase (GPAT), acylglycerol‐phosphate acyl transferase (AGPAT). Dephosphorylation of the glycerol backbone then occurs under the catalysis of phosphatidic acid phosphorylase (PAP), followed by a further acylation of the diacylglycerol by diacylglycerol acyl transferase (DGAT). The TGs produced are packed into very low density lipoproteins (VLDLs) for export from the liver, or are stored within hepatocytes. DH, dehydrogenase; NAD^+^, nicotine adenine dinucleotide; NADH, reduced form of NAD^+^; PDH, pyruvate dehydrogenase; TPI, triose phosphate isomerase.

While glucose is the main substrate for DNL, fructose is a highly lipogenic substrate (Dekker et al., 2010) and this is thought to arise from it bypassing the critical regulatory step catalysed by phosphofructokinase‐1 (PFK‐1) in glycolysis (Basaranoglu, 2013). Fructose is phosphorylated by fructokinase in the liver to fructose 1‐phosphate (F1P) (Hers, 1952). F1P is then the substrate for catalytic cleavage by aldolase, generating dihydroxy‐acetone‐phosphate (DHAP) and glyceraldehyde. Glyceraldehyde is subsequently phosphorylated by triokinase to produce glyceraldehyde 3‐phosphate (G3P) (Mayes, 1993). Thus G3P and DHAP can enter glycolysis (Fig. 2). This has implications that mean fructose may reinforce some of the pathogenic mechanisms leading to NAFLD (Laville & Nazare, 2009; Basaranoglu, 2013), as discussed in Sections IX. and X..

During *de novo* triacylglycerol (TG) synthesis FAs are incorporated through the initial acylation, by acyl‐CoA, of glycerol‐3‐phosphate, generating lysophosphatidic acid (LPA). This step is catalysed by glycerol‐phosphate acyl transferase (GPAT) (Coleman & Lee, 2004). LPA is further acylated by another acyl‐CoA, under the catalysis of acylglycerol‐phosphate acyl transferase (AGPAT) (Aguado & Campbell, 1998), producing phosphatidic acid (PA). This is dephosphorylated by the action of phosphatidic acid phosphorylase (PAP) to generate diacylglycerol (DG). A final acyl‐CoA is used to acylate the DG to a TG utilising the catalytic activity of diacylglycerol acyl transferase (DGAT) (Shi & Cheng, 2008). This process is summarised in Fig. 2.

## III. VLDL PRODUCTION AND ASSEMBLY

Very low density lipoproteins (VLDLs) are complexes containing both lipids and proteins, and serve as the export vehicle for lipids from hepatocytes, hence representing an important fate of lipids produced during hepatic DNL. They are composed of an external monolayer of mostly phospholipids (PLs) and unesterified cholesterol encasing a neutral lipid core (mainly TGs) (Olofsson, Stillemark‐Billton & Asp, 2000; Sundaram & Yao, 2010). On the surface of the VLDLs lies an apolipoprotein B 100 (apoB100) molecule that is essential in the assembly and production of VLDLs (Sparks, Sparks & Adeli, 2012), as well as other apolipoproteins, including C‐III (apoC‐III) and A‐I (apoA‐I). These lipoproteins can range from roughly 35 to 100 nm (Tiwari & Siddiqi, 2012). Several sources indicate that DNL may lead to increased VLDL size (roughly 130 nm) but not the number of VLDL particles secreted (Grefhorst *et al*., 2002; Choi & Ginsberg, 2011).

VLDL production is a two‐step process with initial apoB100 translocation across the endoplasmic reticulum (ER) membrane (Rustaeus *et al*., 1999). During this process it interacts with microsomal triglyceride transfer protein (MTP), which aids the partial lipidation of the nascent apoB100 polypeptide, inhibiting apoB100's degradation (Sparks *et al*., 2012). This partially lipidated apoB100 is the primordial VLDL, or pre‐VLDL, which later forms the VLDL particle proper in the second phase of VLDL biogenesis (Rustaeus *et al*., 1999). This occurs through further lipidation of the apoB100 molecule. It is most widely accepted that the majority of lipidation occurs through fusion with neutral lipid droplets in the ER lumen (Wang, Gilham & Lehner, 2007), which are thought to be formed due to the action of MTP transferring TGs into the ER lumen (Raabe *et al*., 1999; Choi & Ginsberg, 2011). Further to this apoC‐III appears to be crucial for the stabilisation of these neutral lipid droplets, as mutation of their lipid‐binding domain leads to decreased lipid accumulation in the ER lumen (Qin *et al*., 2011).

**Figure 3:**
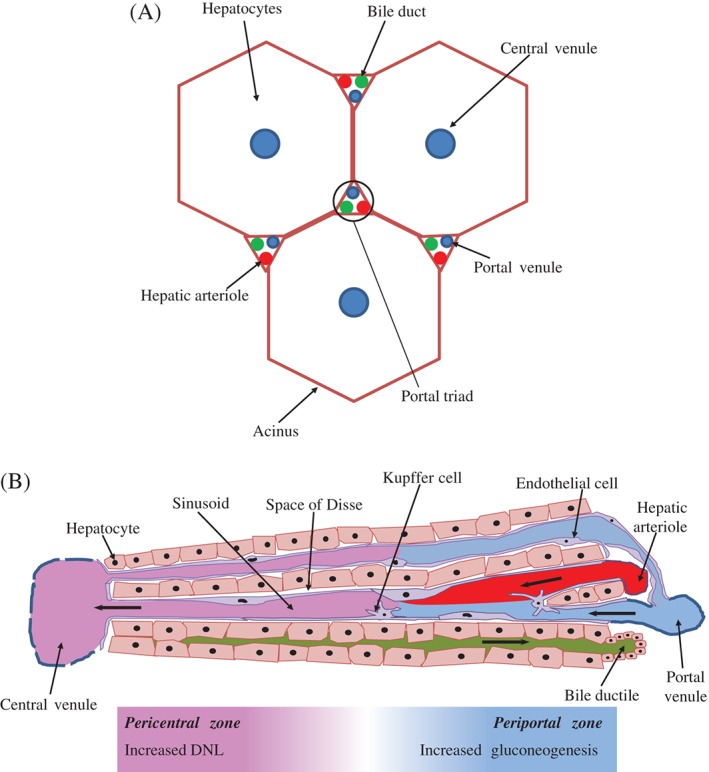
The liver acinus and zonation of metabolic processes. (A) The gross cytoarchitecture of the hepatic parenchyma. Hepatic lobules consist of roughly hexagonal acini with a central vein and portal triads at the interfaces of the acini. (B) A cross section of liver tissue along the portocentral axis, demonstrates the proposed zonation of metabolic processes, with the pericentral zone as the primary site of de novo lipogenesis (DNL) and the periportal zone as the primary site for gluconeogenesis. Endothelial cells make up the walls of the sinusoid which also contains macrophages known as Kupffer cells. As indicated by the arrows, blood flows from the portal area via the sinusoid into the hepatic venule. Bile flows in the opposite direction from hepatocytes to the bile duct through the bile canaliculi. Diagram based on Frevert *et al*. (2005).

From the ER VLDLs are transported to the *cis*‐Golgi by specialised VLDL transport vesicles (VTVs) (Siddiqi, 2008). Within the Golgi the apoB100 is glycosylated and phosphorylated (Swift, 1996; Tran *et al*., 2002). Furthermore, apoA‐I and further lipids may be added to the VLDL particle in its transit across the Golgi, but these aspects are still debated (Tiwari & Siddiqi, 2012). The VLDL particles are then secreted from hepatocytes into the space of Disse (Fig. 3B) *via* fusion of secretory vesicles with the sinusoidal membrane (Alexander, Hamilton & Havel, 1976). From here the VLDLs flow into the circulation.

## IV. THE REGULATION OF HEPATIC DNL

Regulation of the lipogenic pathway is twofold: transcriptional regulation of enzymes integral to FA synthesis, and allosteric regulation of ACC.

The transcriptional regulation of DNL has two major activating pathways: sterol regulatory element binding protein 1c (SREBP1c) and carbohydrate response element binding protein (ChREBP). These two pathways are activated by increased insulin signalling and increased glucose concentrations, respectively; both induced by feeding (Kawano & Cohen, 2013; Oosterveer & Schoonjans, 2014).

### (1) SREBP1c

In the context of DNL, SREBP1c activity is upregulated by insulin signalling *via* an incompletely elucidated pathway. It results in proteolytic release of the active form of SREBP1c from the Golgi membrane, where the membrane‐bound immature form resides, allowing it to translocate to the nucleus, promoting transcription of lipogenic genes. This proteolytic activation of SREBP1c occurs as a result of its interactions with two membrane proteins of the ER, SREBP cleavage‐activated protein (SCAP) and insulin‐induced gene (INSIG) (Ferré & Foufelle, 2010). Upon SCAP changing conformation, either by sterol binding or as a result of phosphorylation events, INSIG dissociates from the SCAP–SREBP1c complex, exposing a MELADL (M = methione, E = glutamate, L = leucine, A = alanine, D = aspartate) amino acid sequence (Sun *et al*., 2007). This sequence then interacts with the coat protein II (COPII) trafficking complex, assisting with the shuttling of the SCAP–SREBP1c complex to the Golgi (Sun *et al*., 2007). Here SREBP1c undergoes a two‐step cleavage by site 1‐protease (S1P) and site 2‐protease (S2P), two proteases present within in the Golgi apparatus necessary for SREBP1c maturation, to be released from the PL membrane in its mature active form (Brown & Goldstein, 2008; Ferré & Foufelle, 2010).

**Figure 4:**
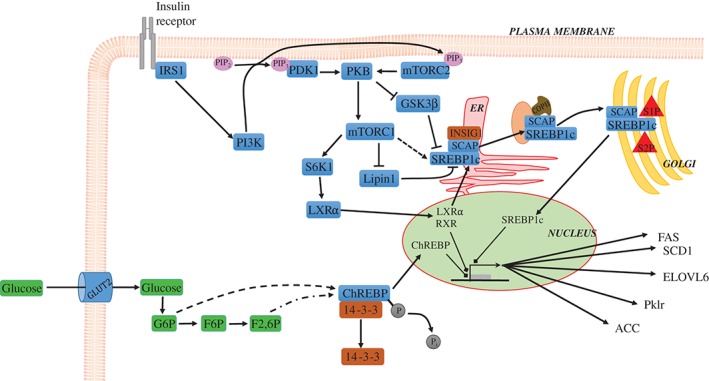
Regulation of de novo lipogenesis by SREBP1c and ChREBP. Insulin activation of the insulin receptor leads to phosphorylation of insulin receptor substrate 1 (IRS1), which subsequently activates phosphoinositide 3‐kinase (PI3K), leading to the phosphorylation of phosphatidylinositol (4,5)‐bisphosphate (PIP_2_) to form phosphatidylinositol (3,4,5)‐trisphosphate (PIP_3_). PIP_3_ activates both phosphoinositide‐dependent kinase 1 (PDK1) and mammalian target of rapamycin complex 2 (mTORC2) (Gan et al., 2011). mTORC2 and PDK1 each lead to phosphorylation of protein kinase B (PKB), with mTORC2 promoting PKB‐mediated inhibition of glycogen synthase kinase 3β (GSK3β) (Hagiwara et al., 2012). PKB also activates mTORC1, leading to activation of ribosomal protein S6 kinase 1 (S6K1) which leads to nuclear localisation of liver X receptor α (LXRα) (Hwahng et al., 2009), heterodimerisation with retinoid X receptor (RXR) and subsequent transcription of lipogenic genes including sterol regulatory element binding protein 1c (SREBP1c). Full maturation of SREBP1c is promoted by the activity of mTORC1 through inhibition of lipin 1 (Peterson et al., 2011), as well as through inhibition of GSK3β by PKB. Both of these events leads to disinhibition of SREBP1c maturation and nuclear localisation. The nascent SREBP1c is a transmembrane protein in the lipid bilayer of the endoplamic reticulum (ER), associated with SREBP cleavage activated protein (SCAP), and insulin‐induced gene 1 (INSIG1). INSIG1 inhibits the SCAP‐mediated shuttling of SREBP1c to the Golgi apparatus via coat protein II (COPII)‐coated vesicles. When SREBP1c and SCAP are phosphorylated or sterols bind to SCAP it changes conformation dissociating from INSIG1 and allowing interaction with COPII coat proteins. SREBP1c is subsequently cleaved by two proteases, site 1‐protease (S1P) and site 2‐protease (S2P), in the Golgi leading to dissociation of SREBP1c from SCAP and removal of the transmembrane domain to allow the mature form of SREBP1c to localise to the nucleus. Once in the nucleus SREBP1c promotes transcription of lipogenic genes including fatty acid synthase (FAS), stearoyl‐CoA desaturase 1 (SCD1), elongation of long‐chain fatty acids family member 6 (ELOVL6), and acetyl CoA carboxylase (ACC). Glucose delivery to the hepatocyte also stimulated the transcription of lipogenic genes via the activation of carbohydrate response element binding protein (ChREBP). This occurs as glucose enters hepatocytes via glucose transporter 2 (GLUT2), and enters the glycolytic pathway. Initially glucose is phosphorylated to glucose 6‐phosphate (G6P), followed by isomerisation to fructose 6‐phosphate (F6P) and further phosphorylation to fructose‐2,6‐bisphosphate (F2,6P) through glycolysis. It has been suggested that both G6P and F2,6P through incompletely elucidated pathways lead to the dephosphorylation of ChREBP and dissociation from the cytosolic protein 14‐3‐3. This allows nuclear localisation of ChREBP and subsequent transcription of its target lipogenic genes, including FAS, SCD1, ELOVL6, ACC and pyruvate kinase, liver and RBC (Pklr).

The activation of SREBP1c occurs *via* two major pathways downstream of the insulin receptor, both involving the phosphoinositide‐3 kinase (PI3K)/protein kinase B (PKB) pathway, one resulting in the phosphorylation of the nascent SREBP1c itself, the other in the activation of the liver X receptor (LXR), predominantly the LXRα isoform in liver (Fig. 4) (Kawano & Cohen, 2013). The use of chemical inhibitors to PKB and PI3K reduced phosphorylation and processing of SREBP1c (Yellaturu *et al*., 2009). Further to this, mammalian target of rapamycin complex 1 (mTORC1) is an integral part of this pathway, being activated when constituently active PKB is expressed in hepatocytes, leading to increased levels of the mature form of SREBP1c and increased rates of DNL (Ricoult & Manning, 2013). This supports the overall role of the PI3K/PKB pathway in insulin‐mediated activation of DNL. Insulin's action *via* this PI3K/PKB pathway thus promotes nascent SREBP1c phosphorylation, leading to increased accumulation of the mature form of SREBP1c, and decreased levels of the membrane‐bound nascent form of the protein in rat hepatocytes and mice *in vivo* (Hegarty *et al*., 2005).

On activation, LXRα heterodimerizes with the retinoid X receptor (RXR) (Chawla *et al*., 2001), which then activates transcription of SREBP1c (Fon Tacer & Rozman, 2011). This is supported by observations in animals lacking LXRα having reduced levels of SREBP1c mRNA, or those treated with synthetic LXRα agonists having increased levels of SREBP1c and resultant lipogenesis (Repa *et al*., 2000). SREBP1c in turn activates DNL through the transcriptional upregulation of several genes involved in FA synthesis, including FAS and ACC (Magaña & Osborne, 1996; Magaña *et al*., 1997).

### (2) ChREBP

ChREBP, in contrast to SREBP1c, is activated by the postprandial rise in glucose delivery to hepatocytes. The rate of glycolysis in hepatocytes in turn increases due to rapid cytosolic equilibration of glucose levels with blood glucose *via* the insulin‐independent glucose transporter family protein, GLUT2 (Mueckler & Thorens, 2013). The activation event of ChREBP appears to be stimulated by a number of metabolites generated during glycolysis, although the exact mechanism is unclear. Glucose 6‐phosphate (G6P) has been proposed to have a role in the regulation of ChREBP (Dentin *et al*., 2012). However, fructose‐2,6‐bisphosphate has also been proposed as a regulator of ChREBP activity in hepatocytes (Fig. 4) (Arden *et al*., 2012).

The main regulation of ChREBP has been suggested to be *via* dephosphorylation of Ser196 and other protein kinase A (PKA) or adenosine monophosphate‐activated kinase (AMPK) phosphorylation sites, which leads to dissociation from 14‐3‐3 protein in the cytosol, translocation to the nucleus and activation of genes containing the carbohydrate response element (ChoRE) (Uyeda & Repa, 2006). However, changes in phosphorylation state may not be the only mechanism of regulation as enzymatic acetylation and *O*‐linked β‐*N*‐acetylglucosaminylation (*O*‐GlcNAcylation), using glucose metabolites as substrates, both increase ChREBP activity (Oosterveer & Schoonjans, 2014). The exact mechanism of regulation is thus still incompletely understood.

The result of this as yet undefined pathway is the upregulation of genes containing the ChoRE, including those genes encoding integral proteins of the DNL pathway such as FAS, ACC and also pyruvate kinase, which provides pyruvate which in turn forms acetyl‐CoA as a lipogenic substrate *via* pyruvate dehydrogenase (Ma, Tsatsos & Towle, 2005). This role of ChREBP has been cemented by experiments in mice lacking ChREBP (Iizuka *et al*., 2004).

### (3) ACC

As discussed above ACC catalyses the synthesis of malonyl‐CoA in DNL, providing the monomer from which FAs are synthesised. It thus provides an efficient control point to limit rates of DNL. ACC is present in two major isoforms in mammals: ACC1 and ACC2 (Bianchi *et al*., 1990). ACC1 is the major isoform in lipogenic tissues such as white adipose tissue, and lactating mammary glands. ACC2 is the prevalent isoform in oxidative tissues such as skeletal and cardiac muscle (Bianchi *et al*., 1990). ACC1 thus acts as the major regulator of DNL in the liver.

The control of ACC1 activity and expression is regulated at a number of different levels. Firstly, ACC is present in low‐activity dimers within cells but it undergoes polymerisation to form higher activity polymers that are filamentous *in vitro* (Ahmad *et al*., 1978) and shown to increase when liver cells are exposed to insulin (Brownsey *et al*., 2006). It has been indicated to be directed by other cytosolic proteins such as midline‐1‐interacting G12‐like protein (MIG12) (Kim *et al*., 2010).

The second level of regulation is *via* allosteric control. Allosteric activators include citrate and glutamate (Boone *et al*., 2000; Brownsey *et al*., 2006). The former promotes the polymerisation of ACC1 to increase its activity, showing feedforward control as citrate is the precursor for acetyl‐CoA production (Brownsey *et al*., 2006). Glutamate is also postulated to increase the polymerisation of ACC (Boone *et al*., 2000), thereby increasing ACC's activity. Allosteric inhibitors of ACC1 include malonyl‐CoA and fatty acyl‐CoA, demonstrating feedback control (Munday, 2001).

The next stratum of control is *via* phosphorylation and dephosphorylation. Phosphorylation occurs rapidly in cells treated with adrenaline or glucagon, leading to inactivation of ACC1 (Brownsey *et al*., 2006). This appears to be mediated mainly through an atypical cAMP–AMPK axis rather than through PKA. The AMPK phosphorylates ACC1 at Ser79, Ser1200 and Ser1215, with the inhibitory effects of AMPK phosphorylation abolished by mutation of Ser79 (Ha *et al*., 1994). PKA does show potential to phosphorylate ACC at Ser77 and Ser1200 *in vitro* and so may have some function *in vivo* (Munday, 2001). In addition glutamate has been shown to increase the activity of type 2A protein phosphatase (PP2A), which leads to removal of phosphate groups from ACC, and increased activity of ACC (Gaussin *et al*., 1996; Boone *et al*., 2000). However, phosphorylation of ACC may not be limited to inhibitory effects and may play a role in insulin‐mediated activation of ACC. Full activation of ACC is accompanied by an insulin‐activated kinase phosphorylation event that is reliant on the PI3K signalling cascade, as inhibition of this pathway reduces insulin‐mediated ACC activation (Brownsey *et al*., 2006).

The final level of control is the regulation of ACC expression, which is under the control of SREBP1c, ChREBP and LXRs in tandem. This is in line with the regulation of expression of other lipogenic enzymes highlighted above.

## V. DNL: A CONTRIBUTOR TO NAFLD

Increased rates of DNL appear to be intertwined with a variety of metabolism‐centred diseases; the most prevalent of these is NAFLD (Kawano & Cohen, 2013). NAFLD is defined as evidence of hepatic steatosis, either by histological section or using imaging, with no causes, such as substantial alcohol intake, steatogenic medical prescriptions or congenital metabolic disorders (Chalasani *et al*., 2012). It is, however, not a well‐delineated disease state but more a spectrum from benign mild steatosis to fibrotic hepatic inflammation, known as non‐alcoholic steatohepatitis (NASH) (Ludwig *et al*., 1980), which may develop into cirrhosis (Masuoka & Chalasani, 2013).

This hepatic accumulation of TG appears to be partly due to lipolytic products of hypertrophied adipocytes (Lewis *et al*., 2002). However, the lipogenic pathway plays an important role. Using labelled palmitate to track FA flux, labelled glyceryl‐tripalmitin to track TG from the diet and labelled acetate to track products of DNL, Donnelly *et al*. (2005) were able to conclude that 59% of TGs in the livers of patients with NAFLD were from FA flux (possibly from lipolysis in adipocytes), 26% from DNL and 15% from the diet. This is supported by data analysing the FA composition of TGs in subjects with and without NAFLD, showing increased levels of saturated FAs in subjects with NAFLD (2009a, 2009b), pointing toward DNL as the source, due to the major product of DNL being saturated FAs. This is consistent with the increased inclusion of DNL‐derived TGs in VLDLs produced in NAFLD, with roughly 15% of produced TG derived from DNL (Diraison, Moulin & Beylot, 2003), compared to 2–5% in normal subjects consuming a typical Western diet (Diraison, Pachiaudi & Beylot, 1997). This is also true in carbohydrate overfeeding in healthy subjects, where DNL‐derived TG made up ∼20% of secreted VLDL TG content (Aarsland, Chinkes & Wolfe, 1996). Thus, in both diseased and healthy states, the contribution of DNL‐derived TG is still the minority of total TG content but correlates with the overall secretion of VLDL (Lewis *et al*., 2002) and may be a marker or regulator of relative FA esterification *versus* oxidation (Schwarz *et al*., 1995), an important consideration in NAFLD which is based on an imbalance of FA oxidation and accumulation.

Further to these measurements of the products of DNL in NAFLD, quantification of transcriptional data for SREBP1c, FAS and ACC1 shows elevation in these crucial regulators and enzymes of DNL in patients with NAFLD (Higuchi *et al*., 2008). The role of SREBP1c in the pathogenesis of NAFLD is further supported by mouse studies where the transcriptionally active form of SREBP1c is expressed in a liver‐specific manner and leads to a consequent accumulation of hepatic lipid droplets (Knebel *et al*., 2012). Further to this, deletion of SREBP1c decreases the TG accumulation by ∼50% in *ob/ob* mice (Moon *et al*., 2012), a mouse model which is hyperphagic and develops severe hepatic steatosis (Zhang *et al*., 1994). Moreover, deletion of SCAP (the escort protein for immature SREBP1c) appeared effectively to prevent TG accumulation in *ob/ob* mice despite maintained hyperphagia and elevated body masses (Moon *et al*., 2012). This promotion of DNL may have further ramifications, as another isoform of ACC, ACC2, is thought to be more functionally orientated towards inhibiting FA oxidation. Evidence suggests that it localises to the outer mitochondrial membrane (Abu‐Elheiga *et al*., 2000) producing malonyl‐CoA that inhibits carnitine palmitoyltransferase 1 and thus the translocation of FAs into the mitochondria for the initiation of *β*‐oxidation (Ruderman, Saha & Kraegen, 2003). This reduces the clearance of FAs through oxidation, further increasing fat accumulation in hepatocytes.

The prevalence of NAFLD, and the role of DNL in this disease, varies widely depending on the population studied and the method employed to assess hepatic steatosis. In the healthy population NAFLD appears to be most prevalent in Hispanic populations (45%), lower in Caucasians (33%) and lower still in African Americans (24%) (Browning *et al*., 2004). Modality of measurement also plays a significant role, as simple analysis using elevated aminotransferases obtains a prevalence of 7–11% (Vernon, Baranova & Younossi, 2011), whereas the use of the gold standard liver biopsy and histological section on potential live donors gave a prevalence of up to 51% for NAFLD (Lee *et al*., 2007). Overall prevalence seems to be increasing in both obese and non‐obese populations, consistently being much higher in the obese population (Cabezas, Mayorga & Crespo, 2012). Thus, NAFLD poses a huge burden on the populus of the developed world, especially considering its association with other metabolic disturbances.

## VI. NAFLD AS A COMPONENT OF METABOLIC SYNDROME AND TYPE 2 DIABETES MELLITUS

The Adult Treatment Panel III, and the International Diabetes Federation and American Heart Association criteria provide specific definitions for the cluster of phenotypic disturbances known as the metabolic syndrome, which includes any three or more of the following: (*i*) hypertriglyceridaemia; (*ii*) low serum high‐density lipoprotein (HDL)–cholesterol concentrations; (*iii*) hypertension; (*iv*) elevated fasting glycaemia; and (*v*) enlarged waist circumference (Grundy *et al*., 2005; Alberti *et al*., 2009; Masuoka & Chalasani, 2013). NAFLD has been proposed extensively as the hepatic incarnation of the metabolic syndrome (Korenblat *et al*., 2008; Almeda‐Valdés, Cuevas‐Ramos & Aguilar‐Salinas, 2009; Paschos & Paletas, 2009; Vanni *et al*., 2010; Masuoka & Chalasani, 2013). This is supported by strong associations between metabolic syndrome and NAFLD, whereby roughly 90% of subjects with NAFLD fulfil one feature of metabolic syndrome, and 33% fulfil three aspects, and thus are defined as having metabolic syndrome (Marchesini *et al*., 2003). Further to this, the prevalence of NAFLD in obese patients with metabolic syndrome is 86%, which is significantly higher than that in healthy individuals (Marceau *et al*., 1999), and patients with metabolic syndrome at baseline were more likely to develop NAFLD subsequently, with an odds ratio of 4 (Hamaguchi *et al*., 2005).

It is well established that metabolic syndrome carries an increased risk of cardiovascular disease (CVD) (Wilson *et al*., 2005; Vanni *et al*., 2010). However, NAFLD also acts, independently of the presence of metabolic syndrome, as a predictor of CVD (Paschos & Paletas, 2009; Vanni *et al*., 2010). This includes increased risk of carotid atherosclerotic plaques (Sookoian & Pirola, 2008), and endothelial dysfunction (Villanova *et al*., 2005; Gaggini *et al*., 2013). Another study has suggested that NAFLD may indeed be a better predictor of CVD than metabolic syndrome (Hamaguchi *et al*., 2007).

NAFLD has also been implicated in T2DM pathogenesis, with up to 85% of individuals with NAFLD having pre‐diabetes or T2DM compared to 30% of controls; all were unaware of their condition (Ortiz‐Lopez *et al*., 2012; Gaggini *et al*., 2013). In a study of individuals with elevated fasting glucose concentration the incidence of T2DM was increased in individuals with NAFLD compared to those without NAFLD (9.9% *versus* 3.7%), suggesting an independent role of NAFLD in the development of T2DM beyond initial insulin resistance (Bae *et al*., 2011). In a study of 1950 participants, 566 were found to have NAFLD, with its prevalence increasing from 27% in those with normal fasting glucose levels, to 43% in those with impaired fasting glucose, and 62% in those with T2DM (Jimba *et al*., 2005). Additionally patients with T2DM on average had 80% increased liver fat compared with control subjects, and this dichotomy increased as waist circumference increased (Kotronen *et al*., 2008).

However, there is evidence that suggests the dissociation of hepatic steatosis and insulin resistance based on evidence from both mouse models of insulin resistance and hepatic steatosis, as well as further interpretation of epidemiological data (Sun & Lazar, 2013). This highlights the fact that it is still not entirely clear how the interaction between NAFLD and metabolic syndrome is delineated in terms of cause and effect, and how they relate to the sequelae of metabolic disease (Vanni *et al*., 2010; Hijmans *et al*., 2013; Sun & Lazar, 2013).

## VII. SELECTIVE INSULIN RESISTANCE PROMOTES HEPATIC DNL

In 1992, a different interpretation was proposed of how the paradigm of insulin resistance, hyperinsulinaemia, hyperglycaemia and hypertryglyceridaemia may all be interlinked (McGarry, 1992). The suggestion was that instead of pathology centred on glucose metabolism, perhaps insulin resistance pathogenesis was better understood as a dysregulation of lipid metabolism, an integral part of this original argument being increased rates of hepatic lipogenesis (McGarry, 1992).

While this view focused on peripheral tissues other than the liver as the major sites of insulin resistance, it is now accepted that insulin resistance is associated with alterations in hepatic lipid metabolism (Brown & Goldstein, 2008; Korenblat *et al*., 2008; Li, Brown & Goldstein, 2010; Samuel, Petersen & Shulman, 2010; Moore, 2012; Hijmans *et al*., 2013). Under this adapted hypothesis, the vicious cycle proposed is still eminent with hyperinsulinaemia leading to increased DNL, and increased insulin resistance (Williams *et al*., 2013). This leads to a failure to suppress gluconeogenesis, and sustained hyperglycaemia, stimulating further pancreatic *β*‐cell insulin secretion. This is a highly simplified view of the process, as aspects involving insulin hypo‐synthesis and hypo‐secretion also appear to play a role in the development of overt T2DM (Poitout & Robertson, 2002; Kelpe *et al*., 2003; Galadari *et al*., 2013).

Subsequent to insulin receptor activation, a divergent kinase cascade within the cytosol is activated, involving PI3K and PKB (Guo, 2014). As a result of this signalling cascade forkhead box protein O1 (FoxO1) is phosphorylated. This prevents its translocation to the nucleus and transcription of its target genes, specifically those involved in gluconeogenesis (Jitrapakdee, 2012). On the other hand SREBP1c is activated by insulin signalling, as discussed in Section IV., promoting lipogenic gene transcription (Ferré & Foufelle, 2010). Thus, in a healthy individual insulin stimulates lipogenesis and suppresses gluconeogenesis. This is in contrast to the insulin‐resistant subject, where insulin signalling is disrupted, as insulin still stimulates lipogenesis but suppression of gluconeogenesis in response to insulin is attenuated (Brown & Goldstein, 2008; Chavez & Summers, 2010). This dichotomy is known as selective insulin resistance, and helps to explain how the typical features of T2DM, hyperglycaemia, hyperinsulinaemia and hypertriglyceridaemia, may exist concomitantly (Brown & Goldstein, 2008). In support of thi s view, severe insulin resistance in the setting of lipodystrophy, where adipogenesis is severely impaired, can be considered: these individuals have increased rates of DNL, hepatic steatosis, hypertriglyceridaemia, hyperinsulinaemia and hyperglycaemia (Garg, 2000; Semple *et al*., 2009).

Under the schema of selective insulin resistance, insulin receptor signalling *via* insulin receptor substrate (IRS)/PI3K/PKB/FoxO1 to suppress gluconeogenesis is dysfunctional, but signalling *via* SREBP1c is maintained. Both *ob/ob* and lipodystrophic mice develop severe insulin resistance due to impaired insulin signalling failing to suppress gluconeogenesis and hepatic steatosis (Bray & York, 1979; Shimomura *et al*., 1998). In these mice the FoxO1 pathway is resistant to insulin‐stimulated downregulation, but insulin still stimulates the activation of SREBP1c (Shimomura *et al*., 2000). Consistent with this, insulin resistance is positively correlated with SREBP1c levels and the downstream lipogenic genes it regulates, whereas genes controlling FA oxidation are downregulated in human individuals with hepatic steatosis (Pettinelli *et al*., 2009). Furthermore, insulin signalling in NAFLD leads to increased SREBP1c levels with reduced inhibitory feedback from other mechanisms as well, including AMPK‐mediated inhibition (Kohjima *et al*., 2008). This supports the role of SREBP1c in the development of NAFLD and subsequent insulin resistance.

However, liver‐specific insulin receptor knock out (LIRKO) mice develop severe insulin resistance, but lack the hepatic steatosis, suggesting divergent signalling pathways in the stimulation of lipogenesis and inhibition of gluconeogenesis (Michael *et al*., 2000; Brown & Goldstein, 2008). Inhibition of the PI3K/PKB pathway either through pharmacological agents or gene knockouts leads to a lack of both stimulation of lipogenesis and inhibition of gluconeogenesis on the application of insulin (Leavens *et al*., 2009; Moore, 2012). This suggests the divergence of the two arms of the insulin signalling may be downstream of PI3K/PKB.

In humans mutations in the insulin receptor lead to a similar phenotype as the LIRKO mice, with severe insulin resistance, hyperinsulinaemia and hyperglycaemia, but not elevated levels of serum TGs (Semple *et al*., 2009). However, mutations in PKB in humans lead to insulin resistance in terms of a failure to suppress gluconeogenesis, but with elevated hepatic DNL, with associated hepatic steatosis and hypertriglyceridaemia (Semple *et al*., 2009). This again supports a divergent pathway between gluconeogenesis and lipogenesis in humans, but preceding the level of PKB rather than later in the signalling cascade. Additionally liver‐specific PI3K knock‐out mice demonstrate a divergence of signalling downstream of PI3K, with PKB governing insulin's effects on gluconeogenesis, whereas the atypical protein kinase C isoforms (PKC λ/ζ) appear to convey insulin's action *via* PI3K to SREBP1c (Matsumoto *et al*., 2003; Taniguchi *et al*., 2006).

However, the relationship between these proposed models of selective insulin resistance and other models of hepatic insulin resistance, which is thought to develop as a result of the increased DG concentrations associated with hepatic steatosis, has not been resolved (Monetti *et al*., 2007; Samuel *et al*., 2010). Under this model activation of PKCϵ, which is activated by DGs, was proposed to inhibit signalling between the insulin receptor and IRS1 (Samuel *et al*., 2004, 2010; Savage *et al*., 2006). This is due to expression levels of PKCϵ being elevated in fatty liver, and inhibition of PKCϵ expression under conditions of fatty liver ameliorating the phenotype of insulin resistance (Samuel *et al*., 2010). Yet again, however, there is still an as‐yet undefined role for another possible mechanism of insulin resistance involving ceramides activating PKCζ and thus inhibiting PKB downstream of insulin receptor/IRS signalling, which has been shown in various cell lines (Holland *et al*., 2007; Hajduch *et al*., 2008; Galadari *et al*., 2013). Ceramides may also inhibit PKB action through stimulating its dephosphorylation and inactivation by PP2A (Chavez *et al*., 2003). This currently leaves a rather confused picture of different levels of possible insulin resistance that may occur in separate pathways controlling gluconeogenesis and lipogenesis.

## VIII. ANATOMICAL ZONATION OF THE METABOLIC PROCESSES IN THE LIVER MAY UNDERLIE ‘SELECTIVE’ INSULIN RESISTANCE

Recently an anatomical theory to explain the maintained sensitivity of hepatic DNL to insulin stimulation in the presence of insulin resistance in terms of a lack of suppression of hepatic gluconeogenesis has been proposed (Hijmans *et al*., 2013). Under this proposition glucose and fat metabolism are ‘zonated’ along the portocentral axis of the liver's functional units, the acini (Fig. 3B) (Katz, 1992; Jungermann & Keitzmann, 1996). To understand this, the anatomy of the hepatic blood flow must be delineated, with blood flowing from the portal venule and hepatic arteriole at the boundaries of the hepatic acinus, along sinusoids to flow out of the acinus' central veins (Fig. 3A) (Ishibashi *et al*., 2009). The cells around the portal veins are described as periportal and those surrounding the central vein as pericentral (Jungermann & Keitzmann, 1996; Ishibashi *et al*., 2009). This alternative to the dichotomous intracellular signalling pathway of selective insulin resistance encompasses pericentral DNL and periportal insulin resistance and a subsequent lack of suppression of gluconeogenesis (Hijmans *et al*., 2013).

To support this perspective of a portocentral axis of insulin resistance there is some evidence from the disease progression of fatty liver disease. Although studies of the zonation of hepatic steatosis are limited, there is evidence for zonation of NAFLD in *ob/ob* mice (Wiegman *et al*., 2003; Hijmans *et al*., 2013), and also in the progression of NAFLD in humans, with greater TG deposits in pericentral areas, progressing to involve intermediate cells and then periportal hepatocytes as the disease progresses (Chalasani *et al*., 2008; Debois *et al*., 2009; Hijmans *et al*., 2013). This leads to the hypothesis that there is preferential uptake of free fatty acids (FFAs) from the blood plasma by periportal rather than pericentral hepatocytes *via* a ‘first‐pass’ mechanism (Hijmans *et al*., 2013). These FFAs are released from adipose tissue under the action of hormone‐sensitive lipase into the circulation, and reach the periportal hepatocytes initially and then pericentral hepatocytes later (Bass, 1990). This would lead to greater accumulation of FFA derivatives, such as ceramides, in the periportal hepatocytes which may cause insulin resistance through the mechanisms highlighted above (Hijmans *et al*., 2013).

However, the mechanism proposed relies on PKB acting as a common component of the insulin signalling pathway and this is not corroborated by findings in individuals with PKB mutations who demonstrate selective insulin resistance (Semple *et al*., 2009; Murphy, Carroll & Krebs, 2013). If the zonal distribution of insulin resistance occurs there would also be increased rates of lipogenesis in periportal hepatocytes as well as pericentral hepatocytes when there is hyperinsulinaemia. This is because inhibition of PKB by the proposed mechanism of ceramide‐mediated insulin resistance (Hijmans *et al*., 2013) would allow maintained stimulation of lipogenesis much as in individuals with monogenic mutations in PKB (Semple *et al*., 2009).

## IX. FRUCTOSE, DNL AND HEPATIC STEATOSIS

Fructose has been suggested partly to underlie the recent increased levels of obesity, NAFLD and T2DM observed in the USA population. This is paralleled by a concomitant increase in fructose consumption in this population during the 20th century from roughly 15 g per day in 1900 to 54.7 g in 2008 (Park & Yetley, 1993; Vos *et al*., 2008; Lim *et al*., 2010). This observational correlation hints at a potential role for fructose in the pathogenesis of the phenomenon of metabolic dysregulation that is increasing in prevalence.

One aspect of the metabolism of fructose is that it is cleared from the blood mainly by the liver (Mayes, 1993). The uptake of fructose by the liver is probably through the family of GLUT proteins, mainly through GLUT2 channels but also including the fructose‐specific transporter GLUT5 (Douard & Ferraris, 2008; Karim, 2012). This allows the fructose to be phosphorylated by the highly specific fructokinase (see Section II.), thereby avoiding the regulatory step of glycolysis catalysed by PFK‐1, and enter metabolic pathways that can promote DNL, inhibit FA *β*‐oxidation and potentially inhibit insulin signalling (Lim *et al*., 2010).

Under this paradigm the majority of fructose generates triose phosphates which undergo gluconeogenesis to be stored as glycogen (Tappy & Lê, 2010). However, a large quantity of acetyl‐CoA is also produced that can enter the lipogenic pathway, increasing the rate of DNL assessed using a ^13^C‐labelled acetate intravenous infusion in humans (Parks *et al*., 2008). Furthermore, fructose has been suggested to activate peroxisome proliferator‐activated receptor gamma (PPARγ) coactivator‐1β (PGC‐1β) which acts as a co‐activator of SREBP1c, thus increasing the expression of enzymes crucial to DNL (Nagai *et al*., 2009). This inhibits hepatic FA *β*‐oxidation, as the lipogenic intermediate malonyl‐CoA inhibits carnitine palmitoyltransferase‐1 (CPT‐1) that translocates fatty acyl derivatives into the mitochondrial matrix for *β*‐oxidation (Topping & Mayes, 1972). Additionally, fructose has been suggested to inhibit the transcriptional activities of PPARα, thus reducing the levels of mitochondrial FA oxidative enzymes regulated by PPARα (Dekker *et al*., 2010; Ament, Masoodi & Griffin, 2012). This shift toward lipogenesis over FA oxidation may contribute to hepatic steatosis and hence insulin resistance through mechanisms involving DG accumulation and PKCϵ activation (Lim *et al*., 2010). Fructose is also considered an activator of mitogen‐activated protein kinase kinase‐7 (MKK7) in rat primary hepatocytes, an upstream activator of c‐Jun N‐terminal kinase (Wei, Wang & Pagliassotti, 2005), which is considered also to inhibit insulin signalling (Hirosumi *et al*., 2002).

## X. FRUCTOSE AND THE GUT MICROBIOME

The gut microbiome has come under much scrutiny in recent years due to potential interplay between bacterial overgrowth, intestinal wall permeability and the development of NAFLD (Abu‐Shanab & Quigley, 2010; Russell *et al*., 2013). This has been suggested to occur partially through the effects of lipopolysaccharide (LPS), or endotoxin, released from Gram‐negative bacterial cell walls in the gut acting on Toll‐like receptor 4 (TLR4) (Medzhitov, Preston‐Hurlburt & Janeway, 1997; Abu‐Shanab & Quigley, 2010). The subsequent activation of inflammatory signalling involving nuclear factor κB (NFκB) (Su, 2002), may link endotoxaemia to subsequent progression of NAFLD. This is supported by evidence of TLR4 signalling which has previously been proposed as playing a role in insulin resistance, being activated in the skeletal muscle of insulin‐resistant human subjects (Reyna *et al*., 2008). In addition, TLR4 signalling has previously been demonstrated in cell culture to be stimulated by ceramides (Fischer *et al*., 2007), and by free fatty acids both in mice and cell culture (Shi *et al*., 2006).

In relation to the gut mircobiome, fructose‐feeding‐induced NAFLD in mice can be ameliorated by TLR4‐inactivating mutations (Spruss *et al*., 2009). Moreover, in mice fed fructose with concomitant administration of antibiotics not absorbed by the intestines, hepatic steatosis can be significantly reduced compared to mice solely fed the fructose‐enriched diet (Bergheim *et al*., 2008). In humans this is further supported by the observation of increased levels of TLR4 expression in hepatic tissue and increased plasma endotoxin presence in individuals with NAFLD and increased fructose intake in their diet, which may be explained by an increased permeability of the intestinal wall to endotoxin, stimulating increased TLR4 expression and activation (Thuy *et al*., 2008).

It should be noted that not all gut microbiota promote fatty liver disease; some strains act as ‘probiotics’ reducing the deleterious effects of other gut microflora (Eslamparast *et al*., 2013). This is supported by evidence that butyrate may be the mechanism by which some bacteria inhibit progression from benign hepatic steatosis to NASH, as has been demonstrated in rat models of NAFLD (Endo *et al*., 2013). Therefore, changes in the microbiome may affect an individual's risk of developing NAFLD, progressing to NASH or the metabolic syndrome.

## XI. CONCLUSIONS

1. The role of DNL within NAFLD may prove a fruitful area of research. It appears to be a component of the pathological basis of the hugely prevalent NAFLD, which may be an important and under acknowledged risk factor for T2DM.
2. Future studies should attempt to clarify whether endogenous metabolites or lipids constitute simple and effective biomarkers of this process.
3. It may be that DNL provides a suitable pharmacological target for early intervention to prevent the onset of T2DM. This would prove hugely beneficial in the developed world where the burdens of over‐nutrition are underpinning unprecedented increases in obesity, prediabetes and overt T2DM.
4. It is clear that the pathological basis of T2DM and the metabolic syndrome are not solely based on increased rates of hepatic lipogenesis, but that DNL is a contributor to the overall paradigm of these metabolic diseases.
5. Investigators should not consider the role of DNL in isolation, as an exclusive cause of insulin resistance, NAFLD and T2DM, but as a part of the total pathogenic disturbance that can lead to a failure of insulin secretion and signalling.
